# Supplementary material for: Generation of a white-albino phenotype from cobalt blue and yellow-albino rainbow trout (Oncorhynchus mykiss): Inheritance pattern and chromatophores analysis
Source: PLoS One. 2020 Jan 27;15(1):e0214034. doi: 10.1371/journal.pone.0214034 (PMC6984684; doi:10.1371/journal.pone.0214034)
Supplement: S1 Table — Standard length (cm; upper) and total body weight (Kg; bottom) of juveniles and adult fish used for pituitary comparison analysis. Data are represented by the average ± SD. Numbers between brackets represent the sample size per color phenotype. (DOCX) [file pone.0214034.s001.docx]

**S1 Table. Morphometrical data of samples used in the analysis of pituitary.**

| **Stage** | **Wild-type** | **Cobalt Blue** | **Yellow-albino** | **White-albino** |
| --- | --- | --- | --- | --- |
| Juvenile (4) | 0.17±0.01 20.3±0.7 | 0.15±0.02 20.3±0.7 | 0.20±0.04 20.0±1.2 | 0.23±0.05 20.5±1.6 |
| Adult female (3) | 0.98±0.06 36.5±1.5 | 1.02±0.03 36.5±1.9 | 1.24±0.12 36.2±1.2 | 1±0.16  34.7±1.9 |
| Adult male (3) | 0.33±0.07 24±1.3 | 0.4±0.15 25.0±2.6 | 1.07±0.08 35.5±0.5 | 0.89±0.42 31.1±5.5 |

Standard length (cm; upper) and total body weight (Kg; bottom) of juveniles and adult fish used for pituitary comparison analysis. Data are represented by the average ± SD. Numbers between brackets represent the sample size per color phenotype.
