# Supplementary material for: Generation of a white-albino phenotype from cobalt blue and yellow-albino rainbow trout (Oncorhynchus mykiss): Inheritance pattern and chromatophores analysis
Source: PLoS One. 2020 Jan 27;15(1):e0214034. doi: 10.1371/journal.pone.0214034 (PMC6984684; doi:10.1371/journal.pone.0214034)
Supplement: S2 Table — Gene names, primers sequence, amplicon size in base pairs and GenBank accession numbers of sequences used for primers design. (DOCX) [file pone.0214034.s002.docx]

**S2 Table. Oligonucleotide information used in qRT-PCR analysis**.

| **Gene name** | **Primer sequence (5`-3`)** | **Amplicon** | **GenBank Accession**  **Number** |
| --- | --- | --- | --- |
| ***Growth hormone (gh)*** | Fw: CTACCTGACCGTCGCCAAGT  Rv: TCTCCAGCCCACGTCTACAG | 69 bp | NM001124689.1 |
| ***Prolactin (prl)*** | Fw: CGAGCTCGCCCAAAAGAAGG  Rv: AGGCCAATGGCATGACAGGA | 101 bp | NM001124733.1 |
| ***Deiodinase 1 (deio1)*** | Fw: GCGAAAAGGTAACTATGACCCAGAA  Rv: AGTCCACATGTGCCCTAGGA | 107 bp | XM021603351.1 |
| ***Deiodinase 2 (deio2)*** | Fw: AGGTCAGGAAGCACCGATCA  Rv: CACACCGTAGGCCACGTTAG | 134 bp | BK009182.1 |
| ***Thyroid-stimulating hormone subunit b (tshb)*** | Fw: TGTGGCACCTGCAACACAGA  Rv: TGTTCAGGCCAGGGTATGGG | 112 bp | NM00112453.1 |
| ***Thyrotropin-releasing hormone (trh)*** | Fw: GCAAGAGGCAAAGAACAACCTTTC  Rv: TTGTTTTCTGGCTAGTGTCACTCA | 85 bp | XM021566749 |
| ***Beta-actin (actinb)*** | Fw: GCACTGGTTGTTGACAACGGA  Rv: AACCATCACTCCCTGATGCCT | 117 bp | AF157514.1 |

Gene names, primers sequence, amplicon size in base pairs and GenBank accession numbers of sequences used for primers design.
